# Targeted Therapy for MAPK Alterations in Pediatric Gliomas

**DOI:** 10.4172/2168-975X.S2-005

**Published:** 2015-07-18

**Authors:** AY Truong, TP Nicolaides

**Affiliations:** Department of Pediatrics and Neurosurgery University of California, San Francisco, CA, USA

**Keywords:** MAPK, Pediatric gliomas, Tumorigenesis, Oncogenic mutations

## Abstract

Although the mitogen-activated protein kinase (MAPK) pathway helps promote normal cell development, the pathway is known to contribute to the initiation and growth of many types of cancers. Tumorigenesis can result from mutations in a number of the pathway’s key proteins, including but not limited to RAS, any one of the three RAF kinases, or MEK1/2. Moreover, by discovering and understanding the biology of oncogenic mutations, scientists can develop novel targeted therapies. This review describes the general history of such targeted therapies in the context of pediatric gliomas. We first describe the biology of gliomas and oncogenic mutations in the MAPK pathway and then summarize notable pre-clinical data and clinical trials for these targeted therapies.

## Introduction

Childhood brain tumors (CBTs) are the most common pediatric solid tumor, and among CBTs, gliomas, which arise from glial cells and their precursors, are the most common [1]. The World Health Organization (WHO) categorizes low-grade gliomas (LGGs) as grade I or II and high-grade gliomas (HGGs) as grade III or IV. Both LGGs and HGGs represent a heterogeneous group of tumor subtypes with incidence and survival rates varying significantly between subtypes [2]. For example, pilocytic astrocytoma, a common type of glioma and CBT, represents 17% of all CNS tumors in 0-to-14 year olds and has a overall 10 year survival rate at >96% [3]. Brain stem gliomas, representing approximately 10% of all pediatric central nervous system (CNS) tumors, are also further subdivided and include its most common subtype, diffuse intrinsic pontine gliomas (DIPGs); greater than 90% of DIPG cases die within 2 years of diagnosis [4]. This dichotomy in survivorship is directly linked to the biology of high-grade tumors and their poor responses to current therapies.

A clinical trial was conducted to determine the effect of temozolomide for treating high-grade astrocytoma, a type of glioma. Pediatric patients with anaplastic astrocytoma (AA) and glioblastoma multiforme (GBM) underwent concomitant chemoradiotherapy with temozolomide and external beam radiation therapy, followed by adjuvant chemotherapy with temozolomide. The study concluded that there was no evidence that temozolomide given during radiation therapy and as adjuvant therapy improved 3-year event-free survival (EFS), and temozolomide failed to improve the outcome in children with high-grade astrocytomas [5]. These results lie in stark contrast to temozolomide’s effect in adult patients where temozolomide has been shown to increase survival, albeit modestly [6]. Rather than discrediting temozolomide’s effect in children entirely, this study should be interpreted as a more broad indication that treating pediatric glioma can be different from treating adult glioma. In order to develop a new arsenal of therapies, scientists must first understand the biology and perhaps discover genetic novelties of pediatric gliomas.

## MAPK Alterations

Despite the dismal prognosis of high-grade gliomas, mounting evidence stresses the importance of the MAPK pathway in tumorigenesis, moving research closer to procuring new therapies. Patients with neurofibromatosis type 1 (NF-1), an autosomal dominant inherited genetic syndrome caused by the deletion/inactivation of *NF1*–a gene that codes for neurofibromin1 - a negative regulator of the RAS/mTOR pathway, are predisposed to developing many types of benign and malignant neural tumors [7]. For example, low-grade gliomas of the optic pathway are observed in 15% of children with NF-1 [8]. While the association between NF-1 and cancer is well documented in the literature, there is some controversy surrounding the importance of NF-1 in the tumorigenesis of spontaneous pilocytic astrocytomas. Some researchers have shown that total *NF1* transcript levels are high in pilocytic astrocytomas and that the *NF1* type I and type II expression ratios in pilocytic astrocytomas are similar to ratios in normal brain tissues and HGGs [9]. This data argues against altered *NF1* gene expression and its role in the tumorigensis of sporadic pilocytic astrocytomas. More recently, Schwartzentruber et al. showed that approximately 25% of sporadic pediatric glioblastomas contain inactivating *NF1* mutations [10]. Such evidence may shed some light on cancers thought to be principally driven by MAPK activation, but nevertheless, the discovery of NF-1 signaled the dawn of a new era for NF-1 associated tumors with hyperactive RAS signaling and RAF activation [11].

## BRAF Alterations

**Figure 1:**
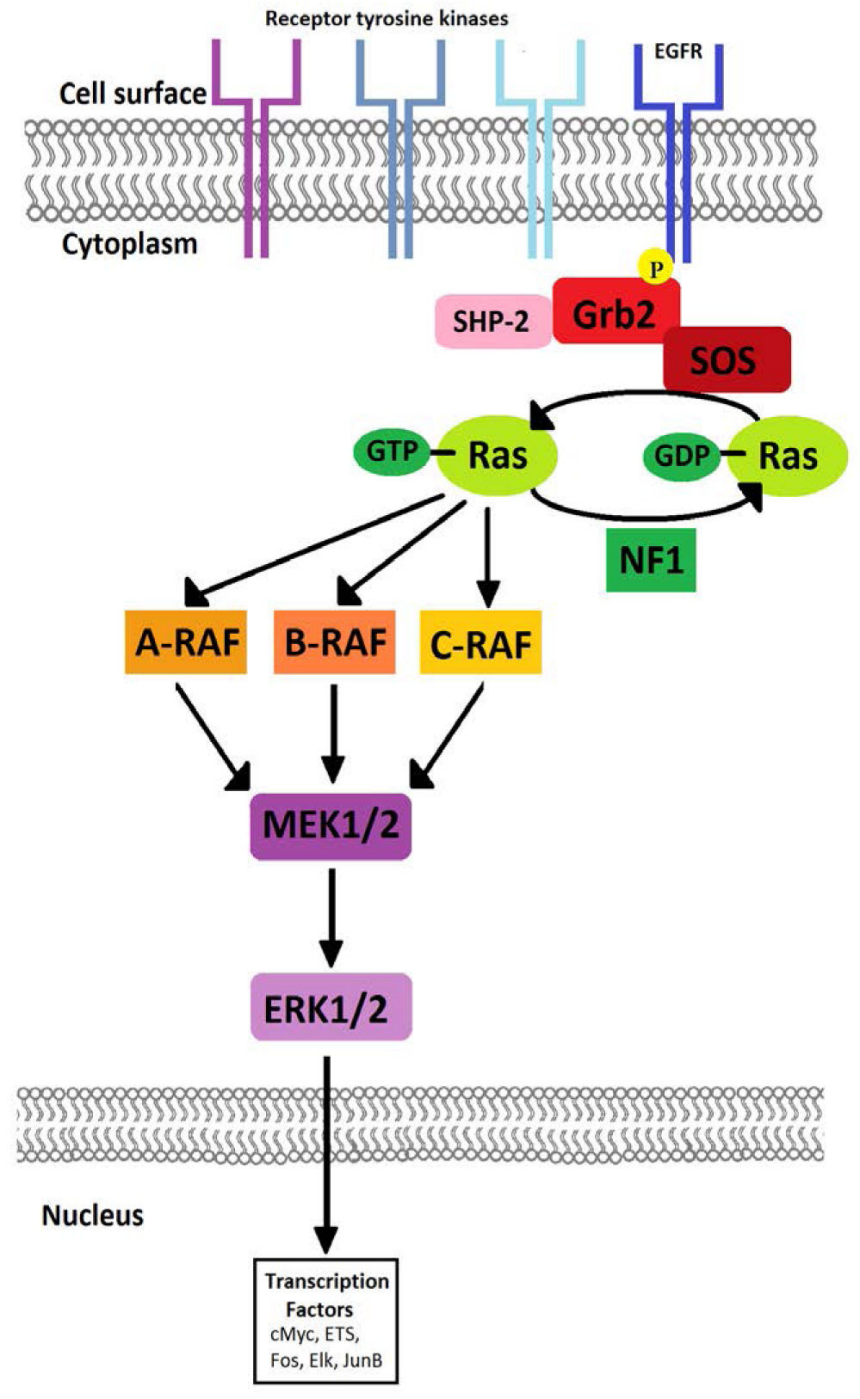
MAPK pathway elements and their interaction.

Perhaps research studying the *BRAF* proto-oncogene is the most promising. As illustrated in Figure 1, BRAF is one of three RAF kinases upstream of MEK1/2. Many mutated BRAF proteins exist, but specifically the oncogenic BRAF fusions and BRAF^V600E^ caused by a missense mutation may prove to be the most prevalent and targetable in pediatric glioma. BRAF fusions, occurring in 66% of pilocytic astrocytomas, were demonstrated to arise from the fusion between *KIAA1549* and *BRAF* as a result of a tandem duplication of ~2Mb at 7q34 [12]. Many studies are trying to elucidate the exact functions of BRAF fusions. Among them is a report that pediatric glioma-associated KIAA1549:BRAF fusions may regulate neuroglial cell growth via the mTOR pathway, depending on cell-type [13]. The finding that NF1 loss and BRAF fusion activation both lead to mTOR activation presents compelling evidence for pairing MAPK pathway inhibitors with mTOR pathway inhibitors. Furthermore, the mechanism leading to mTOR pathway activation is different in each case. NF1 loss activates mTOR, leads to TORC2-dependent AKT activation, and thereby causes p27 phosphorylation and degradation [14]. However, the expression of KIAA1549:BRAF fusion results in MEK-dependent tuberin inactivation and Rheb-directed TORC1/S6K-mediated p27 phosphorylation and degradation [13]. On one hand, these pathways reiterate the complex nature of cancer growth. On the other hand, these driving mutations with their divergent pathways stress the importance of developing targeted therapies catered toward specific mutations in the MAPK pathway itself or toward the pathways leading to or from the MAPK pathway.

In addition, BRAF may also be constitutively activated due to a point mutation converting valine-to-glutamate, creating a BRAF^V600E^ mutant that can activate MEK without first needing upstream RAS phosphorylation [15]. BRAF^V600E^ is one of the most common oncogenic mutations in human cancers, found in approximately 50% of metastatic melanomas, 10% of colon cancer and papillary thyroid cancers [16]. Curiously, BRAF^V600E^ mutant gliomas are most frequently seen in pediatric cases and can be observed in 10% to 75% of all gliomas, varying between the tumor subtypes [17]. For instance, it has been reported that the BRAF^V600E^ mutation is observed in less than 10% of pilocytic astrocytoma and as high as 75% of gangliogliomas [17]. Functioning as both a diagnostic and prognostic biomarker, the BRAF^V600E^ mutation is a good target for proteasome inhibitors [15]. But overall, there have been mixed responses and notable exceptions to point out in regard to the effectiveness of BRAF targeted therapies.

Part of the enthusiasm surrounding BRAF^V600E^ stems from the success of clinical trials using vemurafenib, a small molecule inhibitor targeting BRAF^V600E^, for treating melanoma with the BRAF^V600E^ mutation. In a phase 3 clinical trial comparing vemurafenib versus dacarbazine, Chapman et al. reported that vemurafenib was associated with a relative reduction of 74% in the risk of either death or disease progression as compared with dacarbazine alone [18]. As a result of this and similar studies, vemurafenib and dabrafenib are now FDA-approved for the treatment of BRAF^V600E^ positive melanoma. Similarly for thyroid cancer, Nucera et al. used knockdown studies to conclude that BRAF^V600E^ mutated papillary thyroid carcinoma, is susceptible to selective BRAF^V600E^ inhibitors [19]. Nucera et al. goes further in another manuscript and supports the cause for identifying downstream events from the BRAF^V600E^/ERK1/2 pathway, which may help find novel biomarkers for better thyroid cancer outcome and survival [20]. Yet in stark contrast, there have been many cases where targeting BRAF^V600E^ has not been effective. Using vemurafenib to target colon cancer patients with the same BRAF^V600E^ mutation has shown poor efficacy, which may be due to rapid feedback activation of EGFR. Furthermore, *in vitro* and *in vivo* data suggests that colon cancer cells, unlike melanoma cells that express low levels of EGFR, are particularly prone to this engage in this rescue mechanism. Thus, the authors recommend that BRAF^V600E^ mutant colon cancer with no current targeted treatment options available may benefit from combinatorial BRAF and EGFR inhibitors [21].

A recent clinical trial demonstrated that vemurafenib is only partially effective for patients with advanced papillary thyroid cancer harboring the BRAF^V600E^ mutation. Of the 15 patients enrolled, 7 had a partial response. This is promising, but it is significantly lower than the response rates reported in melanoma patients [22]. Moreover, a manuscript by Montero-Conde et al. proposes a mechanism for thyroid cancer’s resistance to MAPK pathway inhibitors. Their work shows how RAF or MEK inhibitors release transcription repressor CTBP proteins from the HER3 promoter to induce HER3 gene expression. Then, NRG-1 binds to HER3 to trigger HER3/HER2 heterodimerization and receptor phosphorylation, resulting in induced PI3K and reactivated MAPK signaling [23]. Such a mechanism provides the preclinical rationale for why clinicians observe lower incidences of response of thyroid cancers to MAPK pathway inhibitors.

Similarly, a retrospective case study of four adult brain tumor patients with BRAF^V600E^ mutated pleomorphic xanthoastrocytoma (PXA) reported 1 progressive disease response, two stable disease responses, and 1 partial response [24]. As this was a small series of patients, it remains unclear how BRAF^V600E^ mutated PXA and other brain tumors will respond to vemurafenib in the long-term; they may have relatively durable response like melanoma or only transient responses like thyroid and colorectal cancer cells [23]. Despite these findings, there is cause to be optimistic. A recent publication by Robinson et al. reports a complete response of relapsed BRAF^V600E^ mutant pediatric gliomblastoma multiforme to BRAF targeted vemurafenib therapy. The BRAF^V600E^ mutation is rare in glioblastoma multiforme, and the authors suggest that BRAF inhibitors may be particularly effective in pediatric CNS tumors subtypes that express BRAF^V600E^ more prevalently [25]. While there will likely be more mutations discovered that drive MAPK activation, the genetic novelty of BRAF mutations, among other proteins in the MAPK pathway, clearly warrant further exploration.

## Additional Alterations

In a seminal 2013 publication, Jones et al. further delineated the link between *PTPN11*, which encodes the SHP-2 protein, and pediatric glioma. This research revealed new BRAF-activating mutations and recurrent activating mutations in *FGFR1* and *PTPN11* and new *NTRK2* fusion genes in non-cerebellar pediatric low grade gliomas [26]. Overexpression of mutant SHP-2 alone did not elevate levels of phosphorylated ERK in vitro, but the two *FGFR1* mutants, either alone or in combination with mutant *PTPN11*, did lead to upregulation of phosphorlylated ERK. This suggests that *PTPN11*, which is expressed higher in pilocytic astrocytomas compared to normal tissue, may play a role in FGFR1-mutant tumors. Furthermore, all pilocytic astrocytomas in the study’s cohort harbored a MAPK pathway alteration, and Genome MuSiC algorithm suggested that the *BRAF*, *FGFR1*, *KRAS*, and *NF1* were the only genes found to be most significantly mutated. Aside from the *FGFR1* and *PTPN11* mutations, each case usually only possessed one pathway alteration [26]. The research of Jones et al. is important because it furthers our understanding of the mechanisms of tumor growth. While low-grade pilocytic astrocytomas can often be successfully surgically resected, inaccessible midline tumors show slow but chronic disease progression with high morbidity. To treat these types of tumors, physicians may have to rely on targeted therapies, which can be made more effective and specific through the grounded understanding of cancer biology.

The relationship between *PTPN11*, increased RAS/MAPK activation, and brain tumors is complex; presented here is an interesting case of pilocytic astrocytoma that elucidates the intricacies of tumor growth. Approximately 50% of the cases of Noonan syndrome (NS), an autosomal dominant, dysmorphic congenital disorder, are associated with mutations of the *PTPN11* gene leading to RAS/MAPK pathway activation. Although RAS activation is seen in many solid tumors, few NS patients with *PTPN11*-mutations have been reported with solid tumors. Thus, a case report, revealing a patient with both *PTPN11* mutation-associated NS and a pilocytic astrocytoma, is incredibly unique. This report not only describes a novel mutation not previously reported but also implies that the patient’s mutation may represent a more powerful activating mutation given the rare association of these mutations with solid tumors [27].

Similar to the pioneering discovery work of Jones et al., Zhang et al. used whole-genome, transcriptome, and targeted high-throughput sequencing of pediatric low-grade tumors to discover a few genetic alterations including the *KIAA1549-BRAF* fusion, new arrangements and amplifications of *MYB*, and recurrent intragenic duplications of the region of *FGFR1* [28]. According to some scientists, the findings of Jones et al. and Zhang et al. are so powerful that it suggests that, “despite the mutational heterogeneity among different tumors, genetic alterations in LGGs commonly lead to activation of the MAPK-ERK and PI3K pathways”. This would suggest that there is a “degree of biological homogeneity” and that because there is a common mechanism driving cancer growth, there may be there may be a silver-bullet for treating low-grade pediatric gliomas [29]. As a result of their discoveries, researchers like Jones et al. and Zhang et al. catalyzed the current fervor for studying the MAPK pathway.

## Pre-Clinical Data

Despite the number of possible targets in the MAPK pathway, success has been limited. For example, attempts to target mutated, upregulated epidermal growth factor receptor (EGFR), the most common genetic aberration associated with malignant glioma and an upstream component of the MAPK pathway, has been largely unsuccessful due to both inherent and acquired resistance. Possible resistance mechanisms to EGFR tyrosine kinase inhibitors include the acquisition of secondary EGFR point mutations, co-activation and/or amplification of other RTKs, or up-regulation of drug efflux pumps [30]. In general, the resistance mechanisms against EGFR inhibitors are found within the complexities of malignant glioma’s signaling network and many of the mechanisms regulating tumor growth remain to be explained.

However, there has been some recent clinical successes and developments, which are described and aggregated in Table 1. The development of AZD6244 (selumetinib) and PLX4032 (vemurafenib) has led to promising pre-clinical results. AZD6244 is a selective and ATP-uncompetitive inhibitor of the MAPK pathway. It functions by selectively inhibiting MEK1/2, dual-specificity protein kinases that phosphorylate MAPK/ERK kinase 1/2. In vitro data on a panel of tumor cell lines showed that cell lines with *BRAF* or *RAS* mutations were more likely to be sensitive to AZD6244 [31]. In vivo data corroborated these findings and suggested that AZD6244 could inhibit proliferation and induce apoptosis and differentiation in the pediatric BRAF^V600E^ mutant glioma xenograft BT-40 [32].

Similar results have been shown with PLX4720, a BRAF^V600E^ specific small molecule inhibitor. With the subtraction of a phenyl ring, PLX4720 is a tool compound that closely resembles PLX4032, which is FDA approved for treating melanoma with BRAF^V600E^. Mice with orthotopic xenografts created from injected AM-38, a BRAF^V600E^ glioblastoma cell line, showed reduced intracranial tumor growth when treated with PLX4720, but wild-type BRAF intracranial tumors treated with PLX4720 had no survival advantage or delay in tumor growth [33]. Their results indicate a 10% incidence of activating BRAF^V600E^ among pediatric malignant astrocytomas and support the use of BRAF^V600E^ specific inhibitors for patients with the BRAF^V600E^ mutation. As a result of pre-clinical research, selumetinib and vemurafenib moved into pediatric glioma clinical trials.

## Clinical Trials

AZD6244 first entered the clinical trial landscape as a MEK 1/2 inhibitor in two trials. One study compared AZD6244 in combination with docetaxel versus docetaxel alone in *KRAS*-mutated non small cell lung cancer (NSCLC) patients [34]. The other trial is sponsored by the National Cancer Institute and is studying the safety and efficacy of AZD6244 for treating patients with mutated BRAF cancers [35]. In 2014, the results of a phase I study of AZD6244 specifically in children with recurrent or refractory low-grade gliomas showed promising activity. Nine subjects showed sustained partial responses observed after two-to-fifteen courses with four of the tumors having either a BRAF fusion or BRAF^V600E^ mutation, and this drug has now entered a phase II study [36]. All three AZD6244 clinical trials mentioned are currently ongoing.

Aside from selumetinib, other ongoing clinical trials of BRAF^V600E^ mutant cancers include a study involving vemurafenib and a study involving combination therapy of dabrafenib and trametinib. The former trial’s goal is to study the toxicity profile and dose limiting toxicity of vemurafenib in children with recurrent or refractory glioma [37]. The latter trial’s goal is to determine the overall response rate of orally administered dabrafenib and trametinib in subjects with rare BRAF^V600E^ mutated cancers. Dabrafenib and trametinib are both kinase inhibitors that selectively bind to and inhibit BRAF^V600E^ and MEK1/2, respectively. This trial is open to several types of cancers including LGGs and HGGs [38].

In another phase I study with pediatric patients, dabrafenib appears to show promising activity. Patients under 18 years of age with relapsed or refractory BRAF^V600E^ mutation in HGGs, LGGs, Langerhans cell histiocytosis, and other solid tumors were eligible for this study. This is a two-part study. The goal of part one is to determine the recommended phase II dose of dabrafenib based on maximum tolerated dose or systemic exposure similar to that seen in adult patients. In regards to HGGs, investigators reported three complete responses, three partial responses, and two progressive disease responses, and for LGGs, investigators reported eight partial responses, six stable disease responses, and one progressive disease response. Part two of the study is currently underway and involves splitting the patients into four disease-specific cohorts to continue evaluating safety, tolerability, and pharmacokinetics. In addition to being relatively well-tolerated with manageable toxicity, dabrafenib appears to be effective for treating BRAF^V600E^ mutated brain tumors [39].

As a whole, clinical trials of targeted therapy for MAPK alterations have been promising, but a phase II study of children with recurrent or progressive low-grade astrocytomas yielded unexpected results. Sorafenib, a multikinase inhibitor targeting BRAF, VEGFR, PDGFR, and c-kit, was associated with unprecedented acceleration of tumor growth in some subjects. In vitro testing indicated that sorafenib may lead to paradoxical ERK activation in both wild-type BRAF and fusion KIAA1549:BRAF tumor cells as well as NF1-deficient cells, suggesting that BRAF inhibitors should only be used in BRAF^V600E^-mutant tumors [40]. Despite this setback, the study reiterates MAPK pathway’s importance and illustrates the intricacies of the MAPK pathway that have yet to be explained.

## Conclusion

Current scientific research is only beginning to understand MAPK alterations, their effects on tumorigenesis, and how to treat tumors through their targetable mutations. Although small molecule inhibitors are relatively well tolerated, better drugs will be produced. More potent, selective, and effective therapies will be created. Many clinical trials are bound to arise. Some studies, like the sorafenib trial, may reveal unexpected and undesirable results, but with each study, researchers will begin to elucidate the complex mechanisms of the MAPK pathway.

Unlike adult LGGs, lower grade tumors in children, particularly pilocytic astrocytomas, are typically less prone to malignant transformation; furthermore, this may be due to the relatively stable genomes of pediatric LGG, to differing cells of origin for these two cancers, or to other unknown factors [29]. One study suggests that BRAF fusion confers a less aggressive clinical phenotype in pediatric low-grade astrocytoma, which may explain the tendency for growth arrest [41]. However, there is no overwhelming consensus about the relationship between BRAF status and patient survival. The only consensus is that the MAPK pathway plays a physiological and pathological role in the human body, and the MAPK pathway may soon prove to be an even more desirable target for antineoplastic agents than it already is.

## Figures and Tables

**Table 1:** Recent clinical successes and developments of AZD6244 (selumetinib) and PLX4032 (vemurafenib).

Small Molecule Inhibitor	Molecular Target	Patient Eligibility	Clinical Trial Phase
Dabrafenib	BRAF^V600E^	BRAF^V600E^ mutations in HGGs, LGGs, etc.	Phase 1
Vemurafenib	BRAF^V600E^	Children with recurrent or refractory glioma	Phase 1/2
Selumetinib	MEK1/2	Recurrent or refractory low-grade pediatric gliomas	Phase 1/2
Selumetinib + docetaxel	MEK1/2	*KRAS*-mutated NSCLC	Phase 2
Selumetinib	MEK1/2	mutated BRAF cancers	Phase 2
Dabrafenib + Trametinib	BRAF^V600E^ + MEK1/2	LGGs, HGGs, etc.	Phase 2
Sorafenib	Multikinase inhibitor	Low-grade astrocytomas	Phase 2 (Terminated)

## Abbreviations

AA: Anaplastic Astrocytoma
CNS: Central Nervous System
CBT: Childhood Brain Tumor
DIPG: Diffuse Intrinsic Pontine Glioma
EFS: Event Free Survival
EGFR: Epidermal Growth Factor Receptor
GBM: Glioblastoma Multiforme
HGG: High-Grade Glioma
LGG: Low-Grade Glioma
MAPK: Mitogen-Activated Protein Kinase
NSCLC: Non Small Cell Lung Cancer
NS: Noonan Syndrome
PA: Pilocytic Astrocytoma
PXA: Pleomorphic Xanthoastrocytoma
